# Dermal mycobacteriosis and warming sea surface temperatures are associated with elevated mortality of striped bass in Chesapeake Bay

**DOI:** 10.1002/ece3.4462

**Published:** 2018-08-28

**Authors:** Maya L. Groner, John M. Hoenig, Roger Pradel, Rémi Choquet, Wolfgang K. Vogelbein, David T. Gauthier, Marjorie A. M. Friedrichs

**Affiliations:** ^1^ Virginia Institute of Marine Science College of William & Mary Gloucester Point Virginia; ^2^ CEFE UMR 5175 CNRS ‐ Université Montpellier ‐ Université P. Valéry ‐ EPHE Montpellier Cedex 5 France; ^3^ Biological Sciences Old Dominion University Norfolk Virginia; ^4^ Present address: Prince William Sound Science Center 300 Breakwater Ave Cordova Alaska 99574

**Keywords:** climate change, disease, epidemiology, E‐SURGE, incidence, mortality

## Abstract

Temperature is hypothesized to alter disease dynamics, particularly when species are living at or near their thermal limits. When disease occurs in marine systems, this can go undetected, particularly if the disease is chronic and progresses slowly. As a result, population‐level impacts of diseases can be grossly underestimated. Complex migratory patterns, stochasticity in recruitment, and data and knowledge gaps can hinder collection and analysis of data on marine diseases. New tools enabling quantification of disease impacts in marine environments include coupled biogeochemical hydrodynamic models (to hindcast key environmental data), and multievent, multistate mark–recapture (MMSMR) (to quantify the effects of environmental conditions on disease processes and assess population‐level impacts). We used MMSMR to quantify disease processes and population impacts in an estuarine population of striped bass (*Morone saxatilis*) in Chesapeake Bay from 2005 to 2013. Our results supported the hypothesis that mycobacteriosis is chronic, progressive, and, frequently, lethal. Yearly disease incidence in fish age three and above was 89%, suggesting that this disease impacts nearly every adult striped bass. Mortality of diseased fish was high, particularly in severe cases, where it approached 80% in typical years. Severely diseased fish also had a 10‐fold higher catchability than healthy fish, which could bias estimates of disease prevalence. For both healthy and diseased fish, mortality increased with the modeled average summer sea surface temperature (SST) at the mouth of the Rappahannock River; in warmer summers (average SST ≥ 29°C), a cohort is predicted to experience >90% mortality in 1 year. Regression of disease signs in mildly and moderately diseased fish was <2%. These results suggest that these fish are living at their maximum thermal tolerance and that this is driving increased disease and mortality. Management of this fishery should account for the effects of temperature and disease on impacted populations.

## 1 INTRODUCTION

Marine diseases can have lasting effects on populations as well as their associated ecological communities (Aronson & Precht, 2001; Behrens & Lafferty, 2004; Groner et al., 2016; Hoenig et al., 2017). Management of high‐impact diseases requires quantification of their population‐level effects and identification of factors that facilitate transmission, mortality, and morbidity (Groner et al., 2016). This is a challenge for many marine diseases due to the high recruitment variability of hosts across years (Johnson, Grorud‐Colvert, Sponaugle, & Semmens, 2014), challenges associated with tracking and analyzing data on migratory species (Sippel et al., 2015), knowledge gaps about the etiology of many marine diseases (Burge et al., 2016), lack of appropriate diagnostic tools (Burge et al., 2016), and paucity of population‐level data (Groner et al., 2016).

Multistate mark–recapture (MSMR) models are a powerful tool for quantifying the effects of disease in marine species (Cooch et al. 2012). MSMR can be used to estimate population size and transitions rates among sites or disease states while accounting for state‐ or time‐dependence in encounter rates. In addition, these models can yield insight into mechanisms that may be driving epidemiological patterns. For example, it is possible to incorporate environmental and biotic covariates that may drive survival, disease progression, or encounter rates. While mark–recapture is commonly used to estimate population sizes and survival rates of numerous fished species (Pine, Pollock, Hightower, Kwak, & Rice, 2003), or epidemiological processes in terrestrial species (e.g., Graham et al., 2013), it is rarely used to estimate disease processes in marine species (Chaloupka, Balazs, & Work, 2009; Hoenig et al., 2017). Unlike terrestrial systems, recapture of tagged individuals in marine systems is frequently done by commercial or recreational fishermen, who may not be qualified to assess the disease state of a recaptured animal. Multievent MSMR (MMSMR) models are a generalization of MSMR models that can be used to estimate uncertainty or error in specifying the disease state of an individual (Conn & Cooch, 2009). As a result, data that may have been discarded for other analyses can be used to infer disease processes using MMSMR.

Striped bass (*Morone saxatilis*) is a dominant piscivore and comprises an economically and recreationally valuable fishery in the Chesapeake Bay. In the 1990s, striped bass in Chesapeake Bay recovered from a significant population decline associated with overexploitation, environmental degradation, and low recruitment (Richards & Rago, 1999). However, disease is now threatening the health and population viability of the Atlantic coastal migratory stock (ASMFC 2013, Hoenig et al., 2017).

Mycobacteriosis in striped bass was first reported in Virginia in 1997. The disease is typically characterized by granulomatous dermatitis and granulomatous inflammation of the visceral organs, predominantly the spleen and anterior kidney. The etiologic agents of the disease in Chesapeake Bay striped bass are *Mycobacterium shottsii*,* M. pseudoshottsii*,* M. marinum,* and a variety of other undescribed species (Gauthier, Helenthal, Rhodes, Vogelbein, & Kator, 2011; Rhodes et al., 2004). As is typical for infections with *Mycobacterium* spp. in fishes (Colorni, 1992), disease in striped bass develops slowly and some individuals appear to survive for long periods with low‐level infections (Jacobs, Rhodes, Sturgis, & Wood, 2009). Prevalence of dermal mycobacteriosis of fish caught in commercial pound nets can exceed 60% in the Rappahannock River, and recent models suggest that, for fish with severe signs of disease, survival can be 46% lower than in healthy animals (Hoenig et al., 2017). Disease‐associated mortality was previously observed in a study of visceral mycobacteriosis in striped bass from the Chesapeake Bay mainstem (Gauthier et al., 2008). Despite recognition of its potential to cause mortality, quantitative estimates of the population‐level effects of the disease are lacking (Gauthier et al., 2008; Hoenig et al., 2017; Vogelbein et al., 2012). Moreover, it is unknown whether variable capture rates across disease severity states may have biased previous estimates of disease prevalence from a Rappahannock River tag‐recapture study focused on dermal mycobacteriosis (Hoenig et al., 2017), and a Chesapeake Bay mainstem trawl survey focused on splenic mycobacteriosis (Gauthier et al., 2008).

Management of mycobacteriosis in striped bass requires estimates of mortality in healthy and diseased fish, rates of disease progression, and disease incidence as well as conceptual or quantitative models characterizing the influence of environmental factors on these rates. Rates of incidence, disease progression, and recovery have not been estimated. While the effect of disease on survival has been estimated for the Chesapeake Bay, these models have not accounted for potential bias in survival rates due to heterogeneous capture rates (Gauthier et al., 2008; Hoenig et al., 2017). Moreover, the role of environmental factors in modulating disease expression in striped bass is not well understood. Concentrations of *Mycobacterium* species in the water column are positively correlated with temperature, total nitrogen, and total phosphorous and negatively correlated with dissolved oxygen concentrations (Jacobs et al., 2009), suggesting that transmission rates or shedding rates may be environmentally dependent. It has been hypothesized that, during the summer, stressfully warm sea surface temperatures (SSTs) and hypoxia in the bottom waters may “squeeze” striped bass into nonoptimal conditions, which may render them more susceptible to death (Coutant, 1985; Price et al., 1985). It is not known if this squeeze may also impact susceptibility to disease.

In this study, we used MMSMR to estimate survival, progression, and recovery of healthy and diseased striped bass age three and older. Using a 9‐year mark–recapture dataset from the Rappahannock River in the Chesapeake Bay in VA, USA, we evaluated whether these epidemiological variables were correlated with changes in environmental conditions, specifically hypoxia and temperature at the mouth of the river and flow within the river. In order to better understand the consequences of environmental variation and disease on population viability, we used our model output to simulate the effects of different environmental conditions on the population dynamics of a cohort of striped bass across 6 years.

## 2 METHODS

### 2.1 Tagging

Tagging of striped bass took place in the Rappahannock River from September through November every year from 2005 to 2012 and in May for some years. The Rappahannock River was selected as the study site because of the presence of pound nets from which large numbers of striped bass could be tagged with minimal stress due to capture. This river is fairly representative of striped bass habitat in the Chesapeake Bay (Sadler et al., 2012).

**Figure 1:**
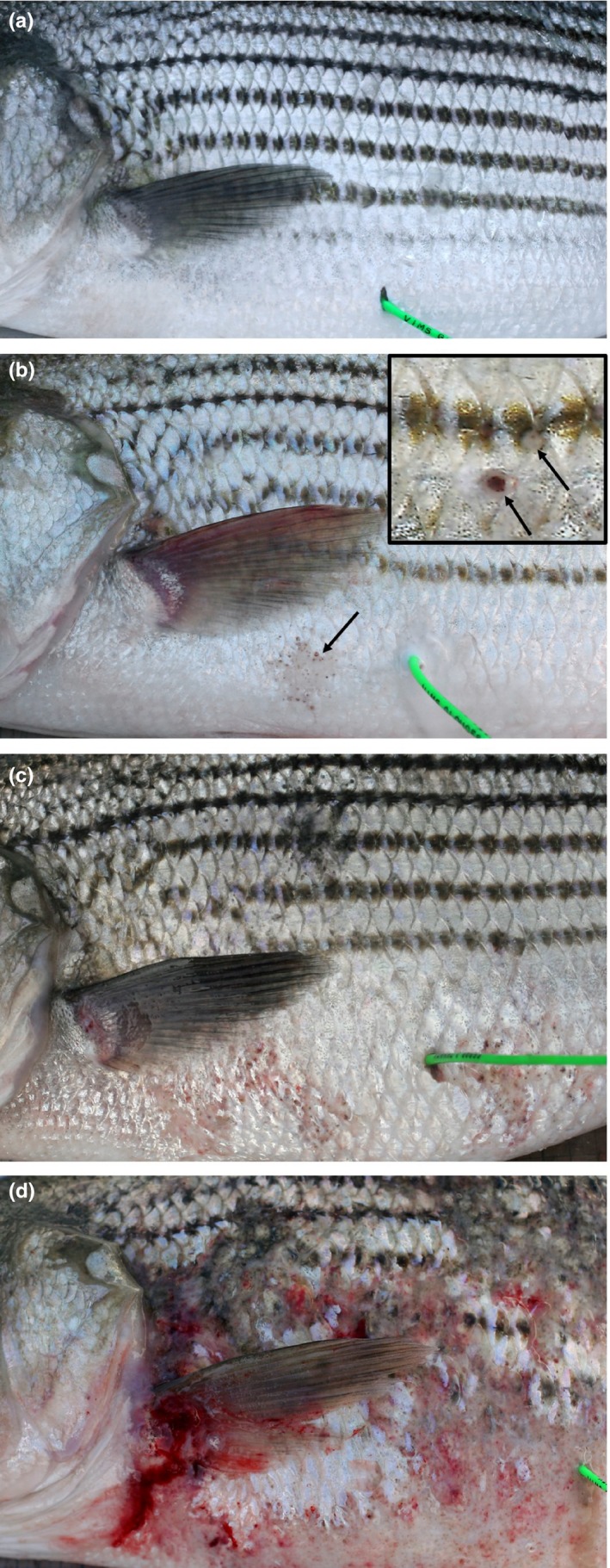
Dermal mycobacteriosis in striped bass, *Morone saxatilis*, from Chesapeake Bay: Externally healthy fish (a), incipient pigmented foci (arrows), and scale loss with mild disease (b), insert depicts higher magnification of two pigmented foci showing central brown pigment and erosion of overlying scale), moderate disease exhibiting up to 50 pigmented foci and small (<2 cm diameter) multifocal early coalescing ulcers (c) and severe dermal disease exhibiting multifocal, shallow hemorrhagic ulceration (>2 cm diameter) with loss of scales and hyper‐pigmentation (d)

Approximately one to three thousand fish were obtained from pound nets at the mouth of the Rappahannock River, Virginia (lat. 37°36.67′N, long. 76°17.49′W) and upriver (lat. 37°58.73′N, long. 76°53.04′W) each year. All fish were greater than 457 mm in total length (minimum legal size), and 95% were between 457 and 610 mm total length which, in the Chesapeake Bay, typically corresponds to between three and 6 years of age. The length of each captured fish was measured. Fish were aged by counting the growth annuli from scales collected during tagging or recapture (Sadler et al., 2012). Previous comparisons of ages of striped bass determined using scales and otoliths show agreement between the two aging methods (Sadler et al., 2012). In addition, both sides of each fish were photographed in order to quantify disease signs at the times of tagging and recapture. Dermal disease status was assessed in photographs using the following classifications (Figure 1):

1. Externally Healthy: no signs of dermal mycobacteriosis;
2. Mild disease: up to 10 pigmented foci per side or a single, small focal skin ulcer (<2 cm^2^) per side of the fish;
3. Moderate disease: from 11 to 50 pigmented foci or multifocal ulcers all less than 2 cm^2^;
4. Severe disease: more than 50 pigmented foci per side or focal or multifocal ulcers greater than 2 cm^2^.

A pigmented focus was a small, external, brown focal lesion appearing as a dot on a fish scale. This was considered the earliest manifestation of the disease. Previous analyses have found a high predictive value between the presence of pigmented foci and skin ulcers and visceral disease in Chesapeake Bay striped bass (positive predictive values of 0.85 and 0.99 respectively, Vogelbein et al., 2012).

Anchor tags were inserted into the body cavity through a small incision into the abdomen; a vinyl streamer remained external to the body with a unique number and a message offering a $20 US reward for return of the fish and a $5 reward for return of the tag. Recaptured animals were obtained from commercial or recreational fishers or by research personnel. Thus, while all fish could be confidently assigned to a disease state at tagging, this was not possible at recapture in cases where the fish were too decomposed to assess the disease state or when the tag, but not the fish was collected. Therefore, in each recapture period, tagged fish were assigned one of the following values: 0 = not recaptured, 1 = ascertained healthy, 2 = ascertained mildly diseased, 3 = ascertained moderately diseased, 4 = ascertained severely diseased, 5 = disease status not ascertained. Fish were handled according to approved IACUC procedures (project assurance number VA‐A3713‐01) and were immediately released back into the water at the tagging location. The research team recaptured fish through 2013. While occasional recaptures from fishermen were reported after 2013, these were excluded from the study due to the low sample size.

We were interested in whether age affected the disease status of individuals, however, very few individuals were captured above 5 years of age and none were <3 years of age. Therefore, we assigned striped bass into one of the three age cohorts, 3, 4, and 5+ years old, based on the scale analysis. Each year these individuals graduated to the next cohort, until they reached the “5‐year and older” cohort. At this point, they remained in that group.

### 2.2 Environmental inputs

Daily estimates of oxygen concentration and temperature were obtained as outputs from a coupled three‐dimensional hydrodynamic‐biogeochemical model of Chesapeake Bay (ChesROMS‐ECB; Feng et al., 2015; Irby et al., 2016; Irby et al. 2018), which is based on the open source community Regional Ocean Modeling System (ROMS). In addition to providing estimates of oxygen, salinity, and temperature, the model contains inorganic and organic carbon and nitrogen state variables including particulate (multiple size classes of detritus, phytoplankton, zooplankton) and dissolved forms (nitrate, ammonium, dissolved inorganic carbon, and dissolved organic matter). ChesROMS‐ECB simulations have been compared extensively with satellite‐derived data (Son, Wang, & Harding, 2014; Werdell et al., 2009) as well as in situ data from the CBP Chesapeake Monitoring Program (Irby et al., 2016). Analyses show that ChesROMS‐ECB has significant success (Stow et al., 2009) in reproducing variability in physical and biogeochemical fields and performs as well or better than other Chesapeake Bay models in terms of reproducing observed temperatures and oxygen concentrations.

Monthly average water flow estimates for the Rappahannock were obtained from the USGS at the most downstream nontidal site on the Rappahannock River (1,668,000 at 38°18′30″, 77°31′46″, USGS 2017). Summer average flow data were estimated by averaging data from July, August, and September.

### 2.3 Multievent multistate mark–recapture

We ran MMSMR models to estimate demographic parameters. The first model step was designed to calculate yearly rates of survival (*ϕ*) for each disease state (healthy, mild, moderate, and severe), and yearly rates of transitions (*ψ*) between healthy and diseased states, conditional on survival. These processes were expressed as the product of two types of transition matrices: the survival and disease status matrices. The “event” matrix was also divided into two matrices. The first matrix (*ψ*) was designed to estimate capture probability of fish in each disease state and the second matrix (*ρ*) was designed to estimate the true state of recaptured individuals that were not assessed at recapture. As many of the recaptures came from fishermen who lethally harvested these fish, we also included a term in the model to indicate whether individuals were right censored (i.e., removed from the study).

We fitted numerous MMSMR models that estimated the effect of the class variables, age, time, and disease state, on yearly rates of survival, state transition, capture, and health assessments. In these models, we tested for all combinations of additive and interactive effects of time, age, and disease state on survival, transitions among disease states and capture rates. We always included an effect of time on capture rates because we had a priori knowledge that sampling efforts differed across years. Full details of the model structure and models that were tested are in the supporting information Table S1. Models were run using the software E‐SURGE (v 2.1.2, Choquet, Rouan, & Pradel, 2009).

### 2.4 Goodness of fit

We ran goodness‐of‐fit tests to check whether there is statistically significant heterogeneity among individuals in the same state and group (i.e., at any point in time). For multistate data, the model typically tested is the JollyMove (JMV) model. It consists of two main components, the 3G test and the M test (Pradel, Wintrebert, & Gimenez, 2003). The JollyMove model allows for time‐ and state‐dependency in the data conditional on the first capture (Choquet, Lebreton, Gimenez, Reboulet, & Pradel, 2009). The 3G test evaluates the hypothesis that “there is no difference in the probability of being reencountered between newly tagged and previously tagged individuals at occasion i in state j.” Our dataset had no instances of multiple recaptures, as fish were lethally sampled at recapture, so the 3G test was not possible. The M test evaluates the null hypothesis that whether or not they are recaptured at time *t*, individuals in the same state at time *t* will not differ in the probability that they are encountered in different states at time *t + *1 and beyond. For our dataset, the test of the JMV model was identical to the M test, because there were no cases of multiple recaptures. Goodness‐of‐fit tests were run using the U‐CARE software (Choquet, Lebreton et al., 2009).

### 2.5 Model selection

We used quasi‐Akaike information criterion for finite sample sizes (qAICc) to compare the fit of all MMSMR models. This consists of correcting the AICc value for overdispersion by dividing the log‐likelihood by the estimated overdispersion coefficient, ĉ. This value is equal to the *χ* ^2^ statistic divided by the degrees of freedom in the group with the highest overdispersion. This conservative approach reduces statistical power to detect an effect, but guards against detection of spurious effects.

When evaluating qAIC values, the models with the lowest values have the most support. When the Akaike weight of the best model is less than 0.9, it is recommended to calculate an average model with coefficients weighted according to their Akaike weight (Burnham & Anderson, 2003).

### 2.6 Environmental correlates of disease processes

Once we determined the best‐fitting MMSMR model, we ran a second set of models in E‐SURGE to determine whether time effects could be better explained by environmental conditions. In this case, we replaced the time effect in the best‐fitting model with different environmental covariates that varied over time. Only those matrices that varied over time in the best‐fitting model could be evaluated for correlations with year‐to‐year variation in summer environmental conditions. Specific environmental metrics tested were (a) the average sea surface temperature (SST) in the summer, (b) the number of days in the summer with SSTs greater than 25°C, (c) the number of days in the summer with sea bottom dissolved oxygen concentrations less than 5.1 mg/L, and (*d*) average summer water flow in the Rappahannock. Summer was defined as the period from July 1 through September 30. The threshold in the second metric was based on the previously determined thermal tolerance for striped bass (Coutant, 1985). Typical thresholds for dissolved oxygen are 3 or 4 mg/L; however, these levels were too infrequent in our data to assess their effects. Therefore, we considered the effects of mild oxygen stress defined by values less than 5.1 mg/L. All environmental variables were standardized prior to model fitting. Single and additive effects of all covariates were tested, with two exceptions: the average SST and the number of days higher than 25°C were not tested in the same model due to lack of independence, and dissolved oxygen and water flow were highly correlated (*ρ* = 0.66, *p* = 0.076) and were therefore not tested in the same model. Analysis of deviance (ANODEV) was used to determine if inclusion of a time‐varying environmental covariate was a significant improvement over a time effect (Grosbois et al., 2008).

### 2.7 Disease prevalence

We were interested in quantifying changes in disease prevalence over time. Estimates from mark–recapture data can be erroneous if capture rates differ between healthy and diseased striped bass. To adjust for this, we first calculated yearly disease prevalence in the fish that were caught in the fall (i.e., the catch prevalence or apparent prevalence). For this calculation, we limited our dataset to fish that were being captured for the first time. Disease prevalence in recaptured fish, which were mostly reported by fishermen, could be additionally biased if fish in one disease state are more likely to survive to recapture than fish in another state. We also calculated disease prevalence for the river (i.e., river prevalence). To do this, we had to correct for the bias in capture rates across disease states. We divided the proportion of individuals in each disease state (the catch prevalence), by the capture probability for that disease state and then scaled these values so that they summed to 1. Values for catch prevalence were calculated for each of the years between 2005 and 2012. There were only four newly tagged striped bass in 2013, so catch prevalence could not be calculated for that year. As estimates of the capture bias could not be calculated for the first year of capture, we could only calculate river prevalence for the years 2006 through 2012.

### 2.8 Population projections

In order to understand how disease and temperature affect striped bass populations, we ran several population projections using the parameters calculated in the previous steps. We simulated a population of 10,000 striped bass (age 3+) over a time span of 6 years. The projections were calculated such that:

n(t+1)=D(Sn(t))

Where *n* is a vector of population size in each of the four states (healthy, mild, moderate and severely diseased), *S* is a matrix of yearly survival probabilities for each state, and *D* is a matrix of yearly transition probabilities between the four disease states. Values for *S* and *D* were obtained from the output of the best‐fitting MMSMR with environmental covariates in the previous step. We assumed there was no immigration or emigration out of this population and no new recruits. The structure of the population matrices is available in the supporting documents.

While it would be more informative to run a simulation of the entire striped bass life cycle, knowledge gaps prevented us from parameterizing all of the required demographic rates. In particular, recruitment and the effects of mycobacteriosis on fecundity are not well quantified.

### 2.9 Data accessibility

All data and code associated with this paper are available on figshare (Groner et al., 2018, https://doi.org/10.6084/m9.figshare.6669275).

## 3 RESULTS

### 3.1 Goodness of fit

We analyzed the capture histories of 19,951 striped bass tagged at 3 or more years of age. There were 1,255 instances of recapture in a subsequent year and no cases of multiple recaptures, as nearly all fish were lethally sampled at the first recapture. The goodness‐of‐fit tests indicated that there was marginally nonsignificant heterogeneity among individuals in the 3‐year age class and significant heterogeneity among individuals in the 4‐year age class (Table 1). There were insufficient data in the 5+ age class (which was comprised of only 14 tagged individuals) to test goodness of fit for this group. In order to account for heterogeneity among individuals in the dataset, we used an overdispersion coefficient (ĉ) of 2.096.

**Table 1:** Results from goodness‐of‐fit test for the JollyMove model

Age class	*χ* ^2^	*p‐value*	Degrees of freedom
3 year olds	21.2	0.058	13
**4 year olds**	**18.9**	**0.026**	**9**
5+ year olds	Not enough data		

### 3.2 MMSMR analyses

Model selection revealed two MMSMR models with Akaike weights greater than 0.1 (Table 2). The second best model only differed from the best model in that it included an additive effect of age on the encounter rate, while the best model did not. Parameter estimates in the weighted averaged model differed from the best‐fit model by an average of <1%. Therefore, for simplicity, we present the best‐fit model below.

**Table 2:** Model selection for MMSMR models examining the effect of age, year, and disease state on survival, transitions between disease states, catchability and assessment uncertainty in striped bass

Model definitions					Model output				
Initial state (*Π*)	Survival (*φ*)	Transition (*ψ*)	Capture (*ρ*)	Assessment (*β*)	np	Deviance	qAICc	∆qAICc	Akaike weight
State_1_	State_1_.Time	State_1_.State_2_	State_1_+Time	State_1_	62	69,802.7	33,419.8	0.0	0.70
State_1_	State_1_.Time	State_1_.State_2_	State_1_+Time+Age	State_1_	63	69,802.3	33,421.6	1.8	0.28
State_1_	State_1_.Time	State_1_.State_2_	State_1_.Time	State_1_	82	69,734.2	33,427.1	7.3	0.02
State_1_	State_1_.[Time+Age]	State_1_.State_2_	State_1_+Time+Age	State_1_	71	69,793.8	33,433.5	13.7	0.00
State_1_	State_1_.Time	State_1_.State_2_	State_1_.[Time+Age]	State_1_	87	69,731.1	33,435.6	15.8	0.00
State_1_	State_1_.[Time+Age]	State_1_.State_2_	State_1_+Time	State_1_	70	69,803.3	33,436.0	16.3	0.00
State_1_	State_1_.[Time+Age]	State_1_.State_2_	State_1_.Time	State_1_	91	69,726.3	33,441.3	21.6	0.00
State_1_	State_1_.[Time+Age]	State_1_.State_2_	Time+Age	State_1_	95	69,735.9	33,453.9	34.1	0.00
State_1_	State_1_.Time	State_1_.State_2_	State_1_.Time.Age	State_1_	114	69,692.6	33,471.2	51.4	0.00

*Notes*

Model definitions include the terms that constrained the matrices associated with initial state, survival, state transitions, catchability, and assessment uncertainty. Each row refers to a unique model. See the appendix for matrix designs associated with initial states (*Π*), survival (*φ*), transitions between disease states (*ψ*), capture rates (*ρ*), and disease assessments at recapture (*β*).

State_1_ and State_2_ represent disease states of striped bass at times *t* and *t *+* *1, respectively. A “.” indicates an interactive effect, while a “+” indicates and additive effect.

Survival of striped bass was affected by the interaction of year and disease state. Thus, the effect of time on survival was not parallel across disease states; however, all across years, survival was highest in healthy striped bass and decreased with increasing disease severity. Averaged across 2006–2013, yearly survival was 0.44 ± 0.13, 0.40 ± 0.09, 0.35 ± 0.07, and 0.13 ± 0.02 (mean ± *SE* of means) in healthy, mildly diseased, moderately diseased, and severely diseased striped bass, respectively. Yearly survival of healthy striped bass ranged from 0.16 ± 0.05 to 1.00 ± 0 (mean ± SE). It ranged from 0.14 ± 0.08 to 0.91 ± 0.23 in mildly diseased striped bass, 0.13 ± 0.06 to 0.71 ± 0.25 in moderately diseased striped bass, and 0.06 ± 0.02 to 0.24 ± 0.10 in severely diseased striped bass. Survival was highest in 2009 and 2013 for all disease states. As survival was affected by time, we conducted additional analyses using ANODEV to quantify the effect of specific environmental covariates (see next section). This slightly changed the survival estimates from those presented above.

**Figure 2:**
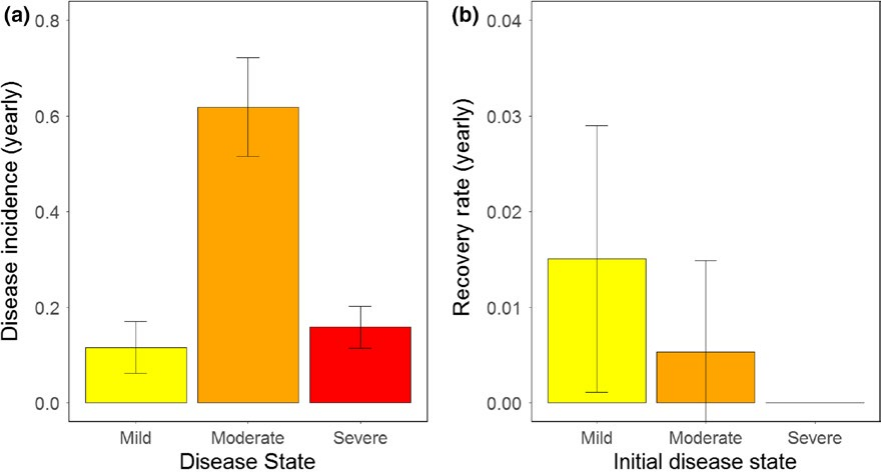
Yearly rates of new cases (disease incidence, a), and recovery from (b) dermal mycobacteriosis in the Rappahannock River from 2005 to 2013 as estimated by the best‐fitting MMSMR model. Note the different scales on the y‐axes. Means and standard errors are shown

**Figure 3:**
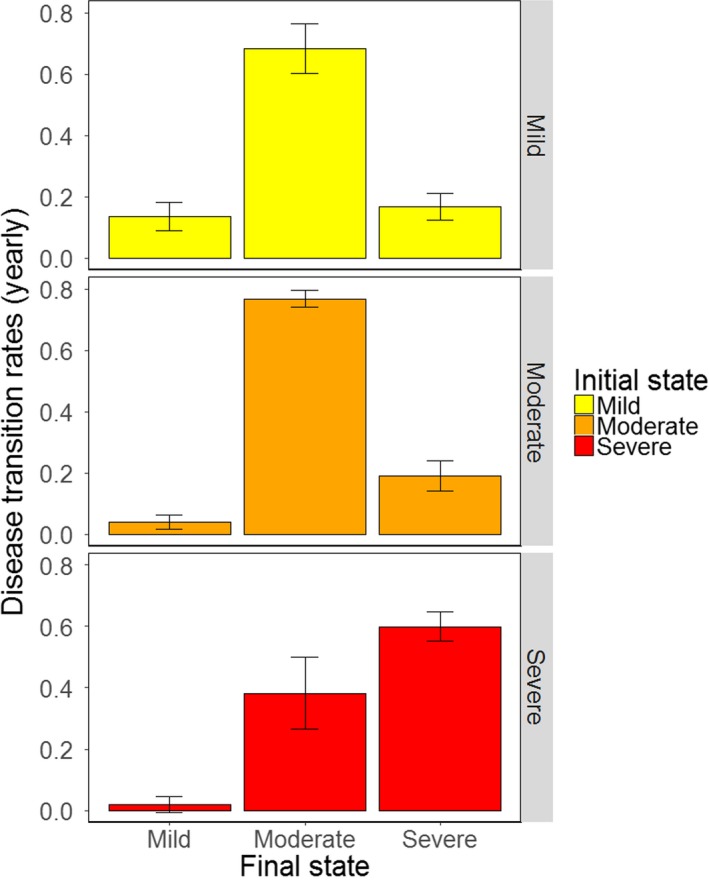
Rates of dermal mycobacteriosis progression (worsening) and regression (improving) in the Rappahannock River from 2005 through 2013 as estimated by the best‐fitting multistate mark–recapture model. Means and standard errors are shown

There was no influence of year on the estimated transition rates. Yearly incidence (the rate that striped bass transition from healthy to diseased) was very high; 89 ± 0.07% of striped bass in this study (age 3 and older) were likely to become diseased. Among these striped bass, the probability of getting mildly, moderately, or severely diseased was 13%, 69%, and 17%, respectively (Figure 2a). In contrast to the high incidence rates, recovery from a diseased state to a healthy state was low (Figure 2b). The yearly probability for recovery was 1.5 ± 0.2% in mildly diseased striped bass, 0.053 ± 0.01% in moderately diseased striped bass, and 0% in severely diseased striped bass. Disease tended to progress over time, with a very low probability of regression (Figure 3). Mildly diseased striped bass had a 13.5 ± 4.6% chance of staying in the same state, a 68.2 ± 8.0% chance of progressing to moderate disease, and a 16.6 ± 4.4% chance of progressing to a severe disease state. Moderately diseased striped bass had a 76.7 ± 2.7% chance of staying in the same state, a 3.8 ± 2.3% chance of regressing to a mildly diseased state, and an 18 ± 4.4% chance of progressing to a severe disease state. Severely diseased striped bass had a 59.9 ± 4.7% chance of staying in the same state, a 1.8 ± 2.5% chance of regressing to a mildly diseased state, and a 38.3 ± 11.8% chance of regressing to a moderately diseased state.

**Figure 4:**
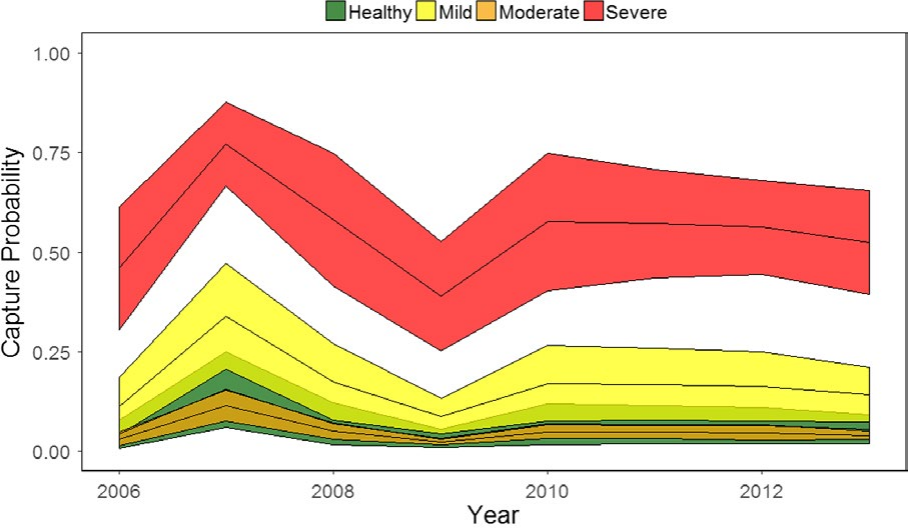
Fall capture rates for healthy striped bass and striped bass with varying degrees of dermal mycobacteriosis from 2006 to 2013. Estimates are from the best‐fitting MMSMR model. Means ± standard errors are shown

Catchability of striped bass depended upon the health of the fish and the year of sampling, but not their interaction (Figure 4). Fish with severe dermal mycobacteriosis had higher catchability than healthier fish. Across years, they had a 55.5 ± 3.9% (mean ± *SE*) chance of being captured, while moderately diseased fish, mildly diseased fish, and healthy fish had 5.1 ± 1.0%, 17.0 ± 2.6%, and 7.0 ± 1.3% chances of being captured in a fall tagging season, respectively. Capture probabilities varied across years. Averaging across disease states, 2007 had the highest capture probabilities (34.6 ± 15.0%) and 2009 had the lowest capture probabilities (13.4 ± 8.6%).

The probability that the health status of a recaptured striped bass could be classified at recapture depended upon its condition at recapture. Striped bass that were healthy, mildly diseased, or moderately diseased at recapture were always assessed. In contrast, only 53.4 ± 1.1% of severely diseased striped bass were assessed. The remainder of these severely diseased striped bass was labeled as having an unknown health status.

### 3.3 Environmental correlates of disease processes

The best model (from the previous section) included an effect of the interaction of time and disease state on survival. We ran numerous models in order to understand if this time effect was better explained by environmental covariates. There was no evidence for time‐dependence in disease incidence, recovery, or transitions among states of disease severity in the best‐fitting MMSMR. Therefore, we did not analyze the impacts of time‐varying environmental covariates on the estimates of these values. While there was an effect of time on capture rates, we did not examine environmental covariates on capture rates because we were less interested in this estimate.

**Figure 5:**
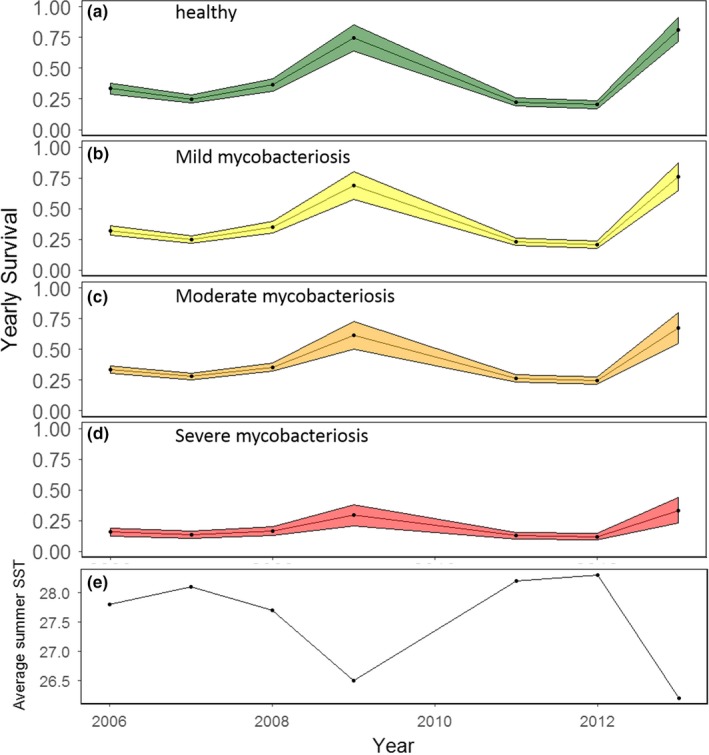
Estimates from the best‐fitting model for survival of striped bass in each disease state (healthy (a), mild (b), moderate (c), or severe (d)) across time. Shading indicates standard errors. The average summer sea surface temperature for the same years is below (e)

**Figure 6:**
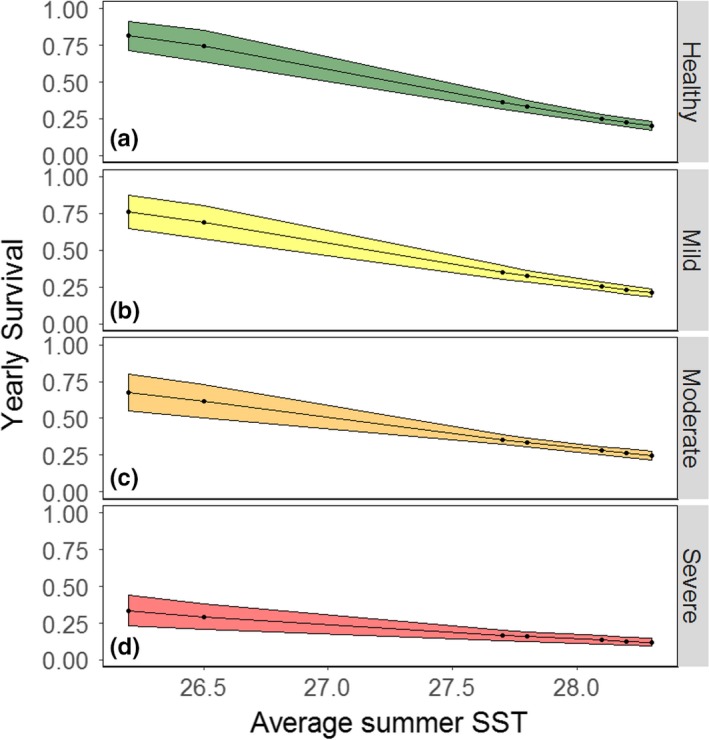
Correlations between sea surface temperature at the mouth of the Rappahannock River and yearly survival for healthy striped (a) bass and striped bass with mild (b), moderate (c), or severe (d) cases of dermal mycobacteriosis between the years 2006 and 2013. Points indicate predicted parameters from the MMSMR model. Predicted effects with standard errors are represented by the black line and shaded band

Analysis of deviance results showed that variations in survival rates for the model with the lowest qAICc were better described with the replacement of the time effect with time‐varying environmental covariates (Table 3, Figure 5). The model that included an interaction for disease state and the average summer SST had the most support, due to its low *p*‐value and the simplicity of the model relative to other models with low *p*‐values. This model showed a negative correlation between increasing average summer SSTs and survival for all states (Figures 6a‐d). Survival was not considerably different between healthy striped bass and mildly or moderately diseased striped bass. In these cases, survival was predicted to be ~75% at 26°C (average summer SST) and decreased linearly to ~25% at 29°C. In contrast, for severely diseased striped bass, survival was 36% at 26°C (average summer SST) and decreased linearly to 9% at 29°C. The range of average estimated summer SSTs during the study years spanned over 2°C, between 26.2°C (in 2013) and 28.3°C (in 2012).

**Table 3:** Results from analysis of deviance (ANODEV), evaluating whether models that included time‐varying environmental covariates of survival (*φ*), were a significant improvement in fit relative to a reference model which included an effect of time on survival

Modeled effect on survival	np	Deviance	*j*	*n*‐*j*	*F*_*j*‐4, *n‐j*_	*p*‐value
***φ*** **(State**_**1**_ * average SST)	**38**	**69,865.2**	**8**	**24**	**9.0**	**0.0001**
***φ*** **(State**_**1**_ * (average SST + DO days))	**42**	**69,848.7**	**12**	**20**	**6.0**	**0.0005**
***φ*** **(State**_**1**_ * (average SST + flow))	**42**	**69,851.3**	**12**	**20**	**5.5**	**0.001**
***φ*** **(State**_**1**_ * heat days)	**38**	**69,897.3**	**8**	**24**	**3.9**	**0.014**
***φ*** **(State**_**1**_ * DO days)	**38**	**69,901.6**	**8**	**24**	**3.5**	**0.02**
*φ* (State_1_ * flow)	38	69,926.3	8	24	1.6	0.21

*Notes*

The environmental covariates tested in the various models listed below include the average sea surface temperature in summer (average SST), the number of days that sea surface temperature was over 25°C (heat days), the number of days when the concentration of dissolved oxygen at the sea bottom was below 5.1 mg/L (days hypoxia), and the average summer river flow (river flow). Interactive effects with the disease state (State_1_) were tested for each environmental covariate. Bolded rows indicate cases where inclusion of time‐varying environmental covariates significantly improved the model fit relative to the reference model.

“np”, number of parameters; “*j”*, number of parameters required to describe the relationship between survival and the environmental covariate; *n*, number of survival estimates in the time‐dependent model.

### 3.4 Disease prevalence

**Figure 7:**
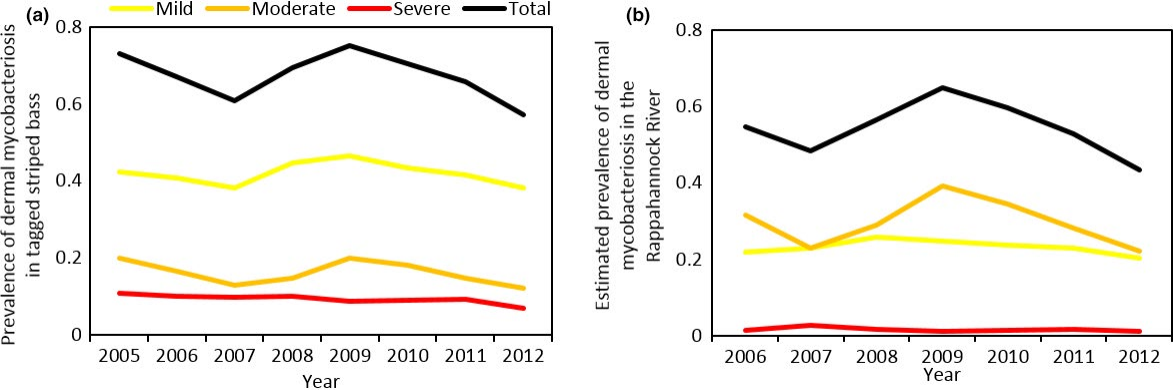
Catch prevalence (the prevalence of dermal mycobacteriosis in striped bass that were tagged) in the Rappahannock River in the fall from 2005 to 2012 (a), and river prevalence (fall prevalence of dermal mycobacteriosis in the Rappahannock River after adjusting for biases in catchability estimated by the best‐fit MMSMR model) from 2006 to 2012 (b)

The catch prevalence of dermal mycobacteriosis in newly tagged striped bass in the fall varied from 57% to 75% in the Rappahannock (Figure 7a). The majority of diseased fish (between 58% and 66% depending on the year) only had mild signs of disease, while 21% to 27% of diseased striped bass had moderate signs of disease and 12% to 16% of striped bass showed severe signs of disease. Data from 16,603 striped bass were used to calculate catch prevalence across years.

After adjusting for bias in capture rates, the estimated prevalence of dermal mycobacteriosis in the river (river prevalence) was slightly lower and markedly less severe than what was found in the tagged animals (Figure 7b). Across years, the estimated prevalence in the Rappahannock ranged from 43% (in 2012) to 60% (in 2010). Among the diseased fish, 38% to 47% were mildly diseased (depending on the year), 47% to 60% were moderately diseased, and 1.6% to 5.3% were severely diseased.

### 3.5 Population projection

**Figure 8:**
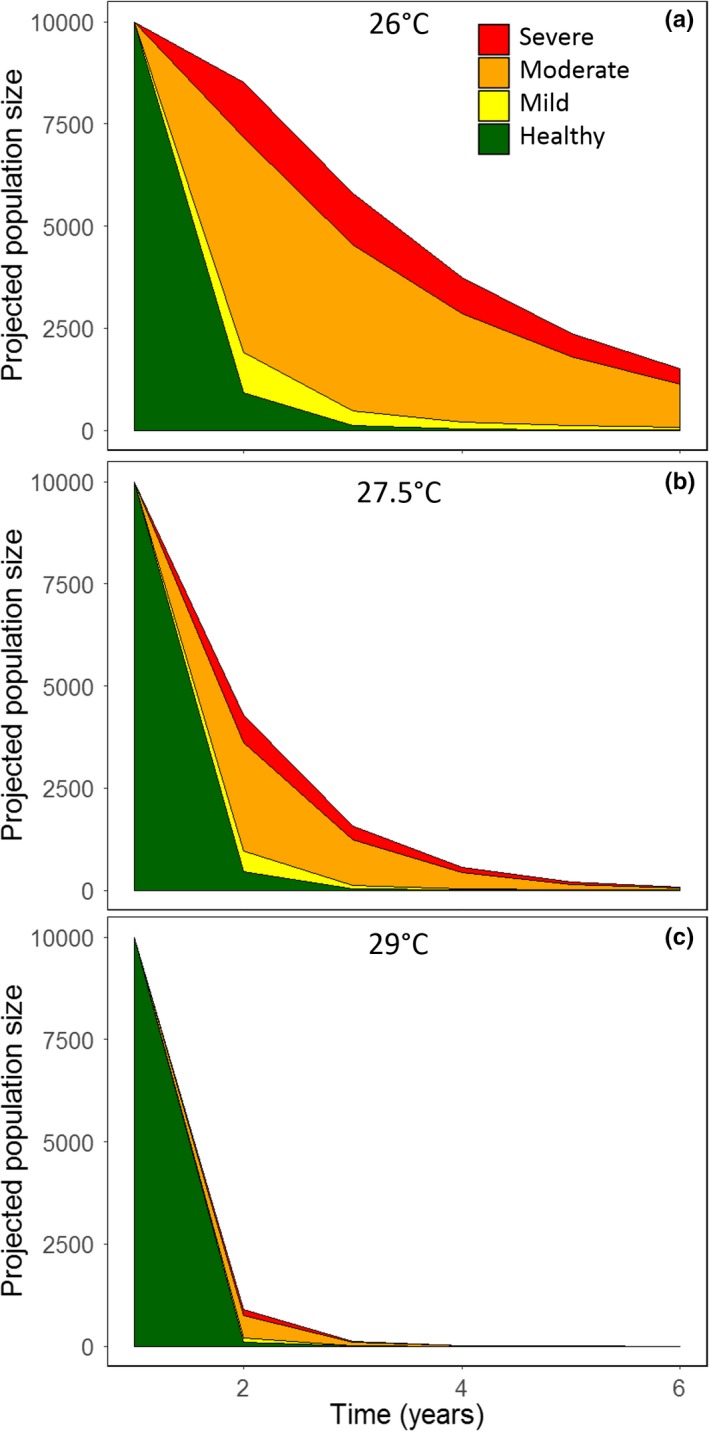
Projected trajectory of population of 10,000 initially healthy striped bass for 6 years when average summer SST is 26°C (a), 27.5°C (b), and 29°C (c) at the mouth of the Rappahannock River

Population projections were conducted using the best‐supported MMSMR model with environmental covariates (described above). As this model included an effect of temperature on survival, we ran projections for mean summer SSTs of 26, 27.5, and 29°C. Population projections of 10,000 striped bass over 6 years indicated that longevity was dependent upon summer SST (Figure 8). When the simulated summer SST was 26°C, 15% of the population was predicted to survive six more years. In contrast, 0.7% were predicted to survive six more years in the 27.5°C scenario, and the population went extinct within 6 years in the 29°C scenario. In all scenarios, the majority of fish were moderately diseased after the first year.

## 4 DISCUSSION

The Chesapeake Bay is the dominant breeding and nursery ground for striped bass on the Atlantic coast (Kohlenstein 1981). While Chesapeake Bay populations of this iconic fish are considered to have recovered since 1995, consistent observations of mycobacteriosis since the late 1990s have raised concerns regarding the stability of this recovery (ASMFC 2013). Our analyses of a 9‐year mark–recapture study of a coastal population suggest that mycobacteriosis and increasing summer temperatures (or factors correlated with summer temperatures) may be increasing mortality in these fish.

Our results support previous hypotheses that mycobacteriosis is a chronic, slowly progressing disease that is highly lethal in advanced stages (Gauthier et al., 2008; Hoenig et al., 2017). Once diseased, progression is likely to continue slowly until the disease is severe, at which point, yearly survival is 36% or less depending upon the average summer SST. As demonstrated by the population projections, this means that disease can contribute substantially to population declines. It is important to note that visceral disease could not be directly measured in this study because fish were sampled alive. Fish with advanced dermal mycobacteriosis do not appear to have significant alteration of osmoregulatory capability as measured by gill and intestinal Na+‐K+‐ATPase activity (Lapointe, Vogelbein, Fabrizio, Gauthier, & Brill, 2014) and so may be able to tolerate severe external disease in this respect. Visceral disease is characterized by granulomatous inflammation of internal organs, especially spleen and anterior kidney. While overall erythrocyte concentration and oxygen carrying capacity do not appear to be impacted in either dermal or visceral disease (Lapointe et al., 2014), other physiological impacts are less well understood and may contribute to the poor prognosis of this disease.

Our model showed that a very small proportion of mildly or moderately diseased fish (<2% per year) can revert to a healthy state. In cases of tag returns, some fish exhibited re‐epithelialization of pigmented foci and/or ulcers, which in some cases would be recorded as a regression of disease states. Histological data generally showed active granulomatous inflammation with acid fast bacteria underlying these lesions (Lapointe et al., 2014), so rather than a “latent” state as found for human tuberculosis (Flynn & Chan, 2001), this appears to represent a slowed or arrested disease state. To date, no records of spontaneous clearance of mycobacterial infections in the laboratory have been published, and piscine mycobacteriosis is still considered to ultimately be a fatal disease (Gauthier, Rhodes, Vogelbein, Kator, & Ottinger, 2003).

The estimated prevalence of dermal mycobacteriosis in striped bass in the Rappahannock River was remarkably consistent and high from 2006 to 2012, ranging between 40% and 60%. While these estimates are an improvement over previous estimates because they account for differences in catchability across disease states, error in estimates may still occur as a result of inaccurate diagnoses based on visual signs. Further work is required to understand how well Rappahannock River prevalence reflects bay‐wide trends. There was no evidence for effects of time on disease incidence, recovery, or progression. The high disease incidence (89% of striped bass 3 years and older are predicted to become diseased each year), in particular, suggests that the detrimental impact of mycobacteriosis on this population is sustained and substantial.

We did not find evidence of an effect of age on disease incidence, disease progression, or recovery from disease. This result appears to contrast with a previous study in the Chesapeake Bay in which the apparent disease prevalence (i.e., prevalence in sampled fish) increased with age then began to decrease in female fish 7 years and older, likely due to mortality of sick fish in older age groups (Gauthier et al., 2008). Our dataset consisted of so few individuals over the age of four that we had to collapse our data into only three age classes. The lack of mark–recapture data on older fish reduced our power to detect an age effect and this may account for the discrepancy in results.

Previous studies have found complex associations between hypoxia and striped bass survival (Costantini et al., 2008; Coutant, 1985; Price et al., 1985). Hypoxia in bottom waters is hypothesized to have benefited striped bass in the 1990s by forcing prey fish above the oxycline where they were more likely to experience predation (Costantini et al., 2008). Over the long term, however, hypoxia is thought to be detrimental to striped bass by leading to a deficit in prey resources and, when combined with SSTs exceeding the thermal tolerance of striped bass, “squeezing” striped bass into suboptimal conditions (Costantini et al., 2008). We found little support for an effect of dissolved oxygen (DO) concentrations on striped bass survival during the duration of our study. This may be the case because, while the lower Rappahannock can experience hypoxic events (DO < 3 mg/L), during half of the years studied here (2006 – 2009), very few hypoxic events were measured in the location of this study.

The survival of both healthy and diseased striped bass was negatively correlated with average summer SST at the mouth of the Rappahannock. A change in the average summer SST from 26.2 to 28.3°C was associated with a 3‐fold increase in the mortality of healthy fish (from 0.2 to 0.6 per year). This suggests that striped bass are living at the edge of their maximum thermal tolerance in Chesapeake Bay (Coutant, 1990). Indeed, in all years of the study, the average modeled summer SST at the mouth of the Rappahannock was above the estimated thermal tolerance limit of striped bass (i.e., 26°C, Rogers, Westin, & Saila, 1980; Coutant, 1985). SSTs in the Chesapeake Bay have been increasing at rates ranging from 0.5 to 1°C per decade (Ding & Elmore, 2015). Although we did not see an increase in disease occurrence or severity over our 8‐year study, these trends suggest that the impact of mycobacteriosis on the striped bass population is becoming more severe across longer (decadal) scales.

Controlled laboratory studies examining the metabolic performance of healthy and striped bass across a range of dissolved oxygen saturation and temperatures reveal a complex interplay among the three conditions. As with most fishes, elevated temperatures increase demand for oxygen (*S*_crit_) in striped bass, suggesting that elevated temperatures reduce hypoxia tolerance in this species. Aerobic scope and factorial scope (measures of metabolic performance) are both compromised in fish with heavy visceral disease, indicating that severe mycobacteriosis impairs the ability of striped bass to tolerate high temperatures and/or hypoxic conditions (Lapointe et al., 2014). These findings are consistent with observed increases in mortality under high river SST conditions; striped bass may be living at the edge of their thermal and/or oxygen tolerances during summer months in these regions, and heavy mycobacteriosis may reduce the ability of these fish to tolerate these marginal conditions.

While our population projection matrices were simple and just tracked a single cohort over time, the comparison of temperature scenarios demonstrates how much a 1 or 2°C increase in average summer SST would influence this population. Data are not available to predict the effects of mycobacteriosis or rising temperature on fecundity; however, our results suggest that the effects could be dramatic because both stressors result in a loss of older reproductive adults. Truncation of age classes due to disease and environmental stress may have drastic consequences for the population since older fish typically have the greatest fecundity (Longhurst, 2002). Diseased, reproductive male and female striped bass have been observed in the Rappahannock River; however, it is unclear what toll disease may take on the quantity or quality of gametes.

Our study has direct management applications. Before 2013, the ASMFC estimate for natural mortality in striped bass was (0.14 per year), and the estimate for fishing mortality in the Bay from 2005 to 2013 ranged from 0.054 to 0.078 per year (Atlantic States Marine Fisheries Commission, 2013). Since then, estimates of natural mortality have increased to include age‐dependent rates, and in adult fish range between 0.37 per year in 3‐year‐old fish and 0.14 per year in 7+ years old fish (Atlantic States Marine Fisheries Commission, 2013). Our results suggest that these natural mortality estimates are low. While these values are consistent with the mortality estimates found for healthy striped bass during cooler summers (e.g., mortality from fishing and natural causes is estimated at ~0.25 per year for 26.5°C average SST), mortality is much higher in diseased fish and in warmer years. Accounting for additional mortality due to disease and temperature during stock assessments may result in more conservative population trajectories. Moreover, disease‐associated mortality will likely increase with warming temperatures in the Chesapeake Bay, so these changes will be relevant into the future. Continued monitoring of disease in striped bass is advised as, once the disease appears, it becomes endemic. In areas where mycobacteriosis has not impacted coastal populations, surveillance with pound nets should be effective at picking up disease, as severely diseased fish have a higher catchability relative to healthy or less severely diseased fish. While more intensive to assess, our results show that the majority of captured striped bass have mild disease and that the disease prognosis (i.e., increasing disease progression and death) is predictable even from this early stage. As summer temperatures are expected to increase in the Chesapeake Bay, the decline of this temperature‐sensitive species is likely to continue, unless these fish are able to evolve physiological or behavioral adaptations to changing conditions. Evaluation of the scope for a northward expansion of major nursery and breeding grounds will aid in assessing the future of this fish.

More broadly, this study demonstrates the value of MMSMR for quantifying the epidemiology of marine diseases in migratory species. If visible signs of disease are reliable diagnostics, this approach can be employed without knowledge of the disease etiology or the entire life history of the host. Moreover, this approach demonstrates the increasing utility of using coupled hydrodynamic‐biogeochemical models (e.g., also see Santos et al., 2018) to produce environmental forcing information for fisheries modeling studies. Data collection for these analyses can be annexed onto studies where mark–recapture techniques are already being employed. Increased use of this powerful technique may aid in quantifying patterns of disease transmission, recovery, and survival in other species, ultimately, leading to improved management.

## CONFLICT OF INTEREST

None declared.

## AUTHOR CONTRIBUTIONS

JMH, WKV, and DTG contributed to the striped bass data collection and diagnostics. MAMF ran the hydrodynamic model. MLG, JMH, RP, RC, and MAMF contributed to the data analysis and interpretation of MMSMR results. All authors contributed to the writing. MLG and JMH generated the project idea.
