# Supplementary material for: Dermal mycobacteriosis and warming sea surface temperatures are associated with elevated mortality of striped bass in Chesapeake Bay
Source: Ecol Evol. 2018 Aug 28;8(18):9384–97. doi: 10.1002/ece3.4462 (PMC6194296; doi:10.1002/ece3.4462)
Supplement: Supplementary file 1 [file ECE3-8-9384-s001.docx]

The following is available as a supporting information for the following manuscript:

Groner ML, Hoenig JM, Pradel R, Choquet R, Vogelbien WK, Gauthier DT, Friedrichs MAM. 2018. Dermal mycobacteriosis and warming sea surface temperatures are associated with elevated mortality of striped bass in Chesapeake Bay. Ecology and Evolution.

**Supporting information**

Table S1. Details of the pattern matrices MMSMR models (2^nd^ column) and the constraints on these matrices for various MMSMR model runs (3^rd^ column). The initial state matrix is designed to calculate the proportion of individuals in each state at time 1. The survival and disease transition matrices are designed to assess transitions between the following states: healthy, mild, moderate, severe or dead. The capture matrix is designed to assess the catchability of an individual (C_Not captured_, C_Healthy_, C_Mild_, C_Moderate_, C_Severe_) given its state and the assessment matrix is designed to assess the probability that the state of an individual can be ascertained if it is captured (0: not captured, 1: captured and ascertained as healthy, 2: captured and ascertained as mildly diseased, 3: captured and ascertained as moderately diseased, 4: captured and ascertained as severely diseased, 5: captured, but disease status was not ascertained). In accordance with E-SURGE notation, parameters estimated by the model are indicated in the matrices in the 2^nd^ column with a greek symbol or an *. In those matrices, the parameters in each row (including the *) sum to 1. In the third column, each row indicates a set of constraints that were included in a model run. These constraints are given in E-SURGE syntax. ‘To’ indicates that there is an effect of the initial condition in the associated matrix, while ‘from’ indicates that there is an effect of the final condition. Time and age indicate that matrix estimates can vary with time or age. ‘Firste + nexte’ indicate that matrix transitions should be estimated separately for time 1 (firste) and all subsequent time steps (nexte). A ‘.’ Indicates an interactive effect while a ‘+’ indicates an additive effect.

|  | Matrix structure | Interactions tested |
| --- | --- | --- |
| Initial State (*Π*): | \| **Healthy** \| **Mild** \| **Moderate** \| **Severe** \| \| --- \| --- \| --- \| --- \| \| *π_1_* \| *π_2_* \| *π_3_* \| *** \| | To |
| State transitions: Survival (***φ***) | \|  \| **Healthy** \| **Mild** \| **Moderate** \| **Severe** \| **Dead** \| \| --- \| --- \| --- \| --- \| --- \| --- \| \| **Healthy** \| *φ_1_* \| *-* \| *-* \| *-* \| *** \| \| **Mild** \| *-* \| *φ _2_* \| *-* \| *-* \| *** \| \| **Moderate** \| *-* \| *-* \| *φ _3_* \| *-* \| *** \| \| **Severe** \| *-* \| *-* \| *-* \| *φ _4_* \| *** \| \| **Dead** \| *-* \| *-* \| *-* \| *-* \| *** \| | From  From + Time  From . Time  From + Age  From . Age  From + Time + Age  From. [Time + Age] |
| State Transitions: Disease (*ψ*) | \|  \| **Healthy** \| **Mild** \| **Moderate** \| **Severe** \| **Dead** \| \| --- \| --- \| --- \| --- \| --- \| --- \| \| **Healthy** \| * \| *ψ* _12_ \| *ψ* _13_ \| *ψ* _14_ \| - \| \| **Mild** \| *ψ*_21_ \| * \| *ψ* _21_ \| *ψ* _24_ \| - \| \| **Moderate** \| *ψ* _31_ \| *ψ* _32_ \| * \| *ψ* _34_ \| - \| \| **Severe** \| *ψ* _41_ \| *ψ* _42_ \| *ψ* _43_ \| * \| - \| \| **Dead** \| - \| - \| - \| - \| * \| | From . To  From . To . Time  From . To . Age  From .To .Time . Age |
| Events: Capture (*ρ*) | \|  \| **C_Not captured_** \| **C_Healthy_** \| **C_Mild_** \| **C_Moderate_** \| **C_Severe_** \| \| --- \| --- \| --- \| --- \| --- \| --- \| \| **Healthy** \| *** \| *ρ_1_* \| *-* \| *-* \| *-* \| \| **Mild** \| *** \| *-* \| *ρ _2_* \| *-* \| *-* \| \| **Moderate** \| *** \| *-* \| *-* \| *ρ _3_* \| *-* \| \| **Severe** \| *** \| *-* \| *-* \| *-* \| *ρ _4_* \| \| **Dead** \| *** \| *-* \| *-* \| *-* \| *-* \| | Firste + Nexte . Time  Firste + Nexte . [From + Time]  Firste + Nexte . From . Time  Firste + Nexte . [Age + Time]  Firste + Nexte . Age . Time  Firste + Nexte .[ From + Time + Age]  Firste + Nexte . From . [Time + Age]  Firste + Nexte . From. Time . Age |
| Events: Assessment | \|  \| **0** \| **1** \| **2** \| **3** \| **4** \| **5** \| \| --- \| --- \| --- \| --- \| --- \| --- \| --- \| \| **C_Not captured_** \| *** \| *-* \| *-* \| *-* \| *-* \| *-* \| \| **C_Healthy_** \| *-* \| *δ_1_* \| *-* \| *-* \| *-* \| *** \| \| **C_Mild_** \| *-* \| *-* \| *δ _2_* \| *-* \| *-* \| *** \| \| **C_Moderate_** \| *-* \| *-* \| *-* \| *δ _3_* \| *-* \| *** \| \| **C_Severe_** \| *-* \| *-* \| *-* \| *-* \| *δ _4_* \| *** \| | From |

*Population projection matrices*

Survival (**S_T_**) and disease transition (**D**) matrices used in population projections of striped bass. In both matrices, rows refer to the state of striped bass at time *t* and the columns refer to the state of striped bass at *t+1*. Possible states are (in order), healthy, mildly diseased, moderately diseased and severely diseased with dermal mycobacteriosis. The survival matrix is a function of the average summer SST (*T*)

$$\boldsymbol{S}_{\boldsymbol{T}}=\left( \begin{matrix} {logit}^{-1}(36.8-1.34T) & 0 & 0 & 0 \\ 0 & {logit}^{-1}(32.0-1.16 T) & 0 & 0 \\ 0 & 0 & {logit}^{-1}(23.8-0.88T) & 0 \\ 0 & 0 & 0 & {logit}^{-1}(15.5-0.62T)) \end{matrix} \right)$$

$$\boldsymbol{D}=\left( \begin{matrix} 0.107 & 0.015 & 0.005 & 0 \\ 0.116 & 0.136 & 0.038 & 0.018 \\ 0.619 & 0.683 & 0.767 & 0.382 \\ 0.158 & 0.166 & 0.189 & 0.599 \end{matrix} \right)$$
